# On the Controversy between Drs. Kentish and Wood

**Published:** 1808-07

**Authors:** 


### *To the Editors of the Medical and Physical Journal.*

Gentlemen,

Ever accustomed to esteem the medical profession as learned, liberal, and philosophical, I cannot help expressing my regret, not only at the asperities which are passing between Drs. Kentish and Wood, respecting priority of title to a presumed *discovery*; but at the unaccountable want of caution in both those gentlemen, in making use of so unauthorized a term. Indeed, I feel paroxysms of perfect astonishment, when reading of our modern medical discoveries! Are these gentlemen, discoverers in the happy predicament of children, who, because their own optics are closed, and the light excluded, suppose themselves invisible? Or do they pay the profession the compliment, to take it for granted, that all but modern reading is confined to themselves and a few others, who, from sympathy,

 will will keep their secret? The public at large, indeed, and the *illiterati* of the profession, may be thus attracted, with whom newly coined terms, a laboured elegance of phraseology, and imposing *verbiage,* so totally different from, and doubtless so superior to, the style of old fashioned authors, may well pass for new discoveries in the gross. Whilst speaking of that abundant and studied elegance, always so prolific of meaning, no reader of the medical learning of the present day can possibly mistake me, or mis-apply my remarks.

Dr. Wood says, (No. 112, p. 517) " In 1793 I collected a few ideas on the subject; which, together with some general principles, I published in that year. To exemplify one of the said general principles, I used the following words, " We can apply not only with safety, but with the best effect, ardent spirits to a part burned or scalded." To wave any enquiry as to the precision of this exemplification, with what degree of propriety could Dr. Wood afterwards make use of an " *if*" with respect to discovery? It would be extreme ill manners to suppose Dr. Wood unapprized of the perfect facility with which he could *collect ideas and general principles on the subject,* and also ascertain, from old established practice, that ardent spirits might be applied with safety, and with the best effects, to a part burned or scalded.

Dr. Kentish, I fear, being defective in the necessary caution used by his antagonist, will not be found to stand on so secure ground. Without attempting to question the utility of his publication, or the merits and success of his practice, I must be bold enough to insist, that the following question of his (p. 418) is both rash and unfortunate. Dr. Kentish demands—" Had the ideas of Heister, or of Sydenham, produced any plan or system, which philosophy could regard, as dictated by common sense, until the period when I wrote my Essay?" I shall answer, without hesitation, they had, and that a practice in the case, equally successful with that of Dr. Kentish, has in consequence prevailed in this country for more than a century. To begin with Dr. Harris, a very eminent and judicious practitioner, his success was invariable in the treatment of scalds and burns, even in the eyes, with camphorated spirit. Dr. James recommended the same practice, which may also be found in various publications during the last century, in some of which oil of turpentine is recommended. This system I have myself found *consistent with common sense,* and such as *philosophy could regard,* whence I advised vised the practice in one of my publications, ten or twelve years since, but at no rate as a novelty. The emollient is obviously often a needful auxiliary to the spirituous plan. The propriety of internal stimulants must surely depend both on the state of the disease and the nature of the patient's constitution; and cases may easily be supposed, in which the exhibition of internal stimulants must be deemed most temerarious practice; opposite stages of the disease will easily occur, in which such internal remedies will be the first to present themselves to the recollection of every man of medical experience.

The application of cold water to an inflamed surface, although likewise no new discovery, has certainly far greater claims to novelty of practice than the stimulant plan; and the only thing worthy of remark upon the whole of the subject is, that it could possibly afford matter for ten lines of controversy. The philosophy of the subject, including its relation to gout, lies in a nut-shell; and one is tempted to suppose, that none but bewildered, or highly prejudiced intellects, could possibly miss it.

I shall conclude these few desultory observations with the late claim of Dr. ——— to the discovery of the use of nitre in gangrene, without altogether concurring with him as to the stage of the disease in which he applies it. I would rather he had not insisted upon this as a new discovery. The purging salt of Glauber was formerly used with the same intent; and to speak from memory, of things long past, I think nitre also, in the French practice. Some four and thirty years since, in a conversation with certain medical friends at Guy's Hospital, the properties of nitre became the subject, and its extensive use at Bartholomew's, which had obtained ten or twelve years previously to that period, and if my memory has not deceived me, as an external application in gangrene. I repeat not this so much with a view to invalidate the claim of the gentleman above alluded to, as with that of giving a seasonable, and I trust you will think, a necessary caution in the assumption of NEW DISCOVERIES.

I am, &c.

PRACTICUS.

